# A GWAS Study on Liver Function Test Using eMERGE Network Participants

**DOI:** 10.1371/journal.pone.0138677

**Published:** 2015-09-28

**Authors:** Bahram Namjou, Keith Marsolo, Todd Lingren, Marylyn D. Ritchie, Shefali S. Verma, Beth L. Cobb, Cassandra Perry, Terrie E. Kitchner, Murray H. Brilliant, Peggy L. Peissig, Kenneth M. Borthwick, Marc S. Williams, Jane Grafton, Gail P. Jarvik, Ingrid A. Holm, John B. Harley

**Affiliations:** 1 Center for Autoimmune Genomics and Etiology, Cincinnati Children’s Hospital Medical Center (CCHMC), Cincinnati, OH, United States of America; 2 University of Cincinnati, College of Medicine, Cincinnati, OH, United States of America; 3 Division of Biomedical Informatics, Cincinnati Children's Hospital Medical Center, Cincinnati, OH, United States of America; 4 Center for Systems Genomics, The Pennsylvania State University, University Park, PA, United States of America; 5 Division of Genetics and Genomics, Boston Children’s Hospital (BCH), Boston, MA, United States of America; 6 Center for Human Genetics, Marshfield Clinic, Marshfield, Wisconsin, United States of America; 7 Genomic Medicine Institute, Geisinger Health System, Danville, PA, United States of America; 8 Group Health Research Institute, Seattle, WA, United States of America; 9 Department of Medicine, University of Washington, Seattle, WA, United States of America; 10 Department of Genome Sciences, University of Washington, Seattle, WA, United States of America; 11 Division of Genetics and Genomics and The Manton Center for Orphan Disease Research, Boston Children’s Hospital, Boston, MA, United States of America; 12 Department of Pediatrics, Harvard Medical School, Boston, MA, United States of America; 13 U.S. Department of Veterans Affairs Medical Center, Cincinnati, OH, United States of America; National Cancer Institute, National Institutes of Health, UNITED STATES

## Abstract

**Introduction:**

Liver enzyme levels and total serum bilirubin are under genetic control and in recent years genome-wide population-based association studies have identified different susceptibility loci for these traits. We conducted a genome-wide association study in European ancestry participants from the Electronic Medical Records and Genomics (eMERGE) Network dataset of patient medical records with available genotyping data in order to identify genetic contributors to variability in serum bilirubin levels and other liver function tests and to compare the effects between adult and pediatric populations.

**Methods:**

The process of whole genome imputation of eMERGE samples with standard quality control measures have been described previously. After removing missing data and outliers based on principal components (PC) analyses, 3294 samples from European ancestry were used for the GWAS study. The association between each single nucleotide polymorphism (SNP) and total serum bilirubin and other liver function tests was tested using linear regression, adjusting for age, gender, site, platform and ancestry principal components (PC).

**Results:**

Consistent with previous results, a strong association signal has been detected for *UGT1A* gene cluster (best SNP rs887829, beta = 0.15, p = 1.30x10^-118^) for total serum bilirubin level. Indeed, in this region more than 176 SNPs (or indels) had p<10^−8^ spanning 150Kb on the long arm of chromosome 2q37.1. In addition, we found a similar level of magnitude in a pediatric group (p = 8.26x10^-47^, beta = 0.17). Further imputation using sequencing data as a reference panel revealed association of other markers including known TA7 repeat indels (rs8175347) (p = 9.78x10^-117^) and rs111741722 (p = 5.41x10^-119^) which were in proxy (r2 = 0.99) with rs887829. Among rare variants, two Asian subjects homozygous for coding SNP rs4148323 (G71R) were identified. Additional known effects for total serum bilirubin were also confirmed including organic anion transporters *SLCO1B1-SLCO1B3*, *TDRP and ZMYND8* at FDR<0.05 with no gene-gene interaction effects. Phenome-wide association studies (PheWAS) suggest a protective effect of TA7 repeat against cerebrovascular disease in an adult cohort (OR = 0.75, p = 0.0008). Among other liver function tests, we also confirmed the previous effect of the ABO blood group locus for variation in serum alkaline phosphatase (rs579459, p = 9.44x10^-15^).

**Conclusions:**

Taken together, our data present interesting findings with strong confirmation of previous effects by simply using the eMERGE electronic health record phenotyping. In addition, our findings indicate that similar to the adult population, the *UGT1A1* is the main locus responsible for normal variation of serum bilirubin in pediatric populations.

## Introduction

Bilirubin is the ultimate product of catabolism of heme with complex interplay of different enzymes. Levels of serum bilirubin are significantly elevated in a number of diseases associated with jaundice and hemolytic blood disorders. Bilirubin levels have a large variability in normal population and bilirubin concentration is under strong genetic regulation with a heritability estimate of roughly 0.50 [1]. In particular, polymorphism TATA box promoter motif in the *UGT1A1* gene promoter (UGT1A1*28, rs8175347) is associated with UGT1A1 expression [2]; the mean bilirubin levels of TA7 homozygotes are approximately double those of TA6 homozygotes [3–5].

This homozygosity predisposes individuals not only to Gilbert syndrome which is a benign form of episodic jaundice [2] but also to hyperbilirubinemia induced by several drugs, such as Indinavir, an HIV protease inhibitor, that competitively inhibits UGT enzymatic activity [6]. In addition, TA7 homozygote individuals are also susceptible to severe neutropenia following the administration of Irinotecan, an anti-cancer drug mainly used in metastatic colorectal cancer patients [7]. In this instance, there is a decrease in the inactivation process of the active metabolite SN-38 by glucuronidation to SN-38 glucuronide (SN-38G) [7]. Apart from classical TA repeat insertion, recent data have also shown that other common variants can contribute to mild hyperbilirubinemia in an additive manner with TA repeat, in particular rs4124874 and a coding SNP in UGT1A1 rs4148323 (G71R), with an influence on expression levels of UGT1A1 [8,9]. However, their minor allele frequencies are variable across populations and therefore lead to varying effects of Irinotecan application depending on ethnicity [10,11]. For example, rs4148323 (G71R) which is almost non-polymorphic in European and African population has a minor allele frequency of about 20% in Asians while TA7 repeat is less frequent in this population (~10%) [12].

Homozygosity for rare pathogenic coding variants on the other hand are responsible for severe deficiency of enzymatic activity that range from lethal hyperbilirubinemia (Crigler-Najjar type 1) with zero enzyme activity to Crigler-Najjar type 2 (CN-2) with very low enzyme activity [13, 14]. These pathogenic variants are extremely rare in the general population.

Founded in 2007, the eMERGE Network is a consortium of multiple adult and pediatric institutions using biorepositories linked to electronic medical record (EMR) systems for large-scale genomic research [15]. As part of this network, we conducted this study in order to test the validity of phenotypic data derived from EMR and the capability of this network for genetic research.

Genome-wide association studies (GWAS) for total serum bilirubin or other liver function tests previously have been reported in different ancestries of well characterized adult populations [16–24]; however, the genetic contribution in a pediatrics population has not been specifically evaluated. There is a tendency toward more extreme phenotypes in pediatrics and some genomic effects may be age dependent or stronger in earlier stage of development than later on. In addition, understanding genomic loci that operate in pediatric population are especially important for a better health care since there is an opportunity for early preventive measures as well as long term implementation of a treatment plan in order to avoid further hepatotoxicity and hyperbilirubinemia. Finally the joint effect model of pediatric and adults will provide a better estimate of percentage of variance explained. In this study, we investigate whether susceptibility loci identified by GWAS in adults are associated in children and adolescents. For this purpose, we conducted quantitative GWAS analyses for liver function tests in a primarily European-derived population and compared the observed size effects between pediatric and adult participants from the eMERGE Network.

## Materials and Methods

### Ethics statement

The eMERGE Network is a collaboration of institutions with biobanks linked to EMRs. The detail of recruitment and biological sample collection of eMERGE cohorts has been described previously [15]. De-identified samples linked to EMR were supplied from multiple investigators from different institutions with approval from their respective institutional review boards (IRBs) including Cincinnati Children’s Hospital Medical Center, Boston Children’s Hospital, Geisinger Health System, Group Health Research Institute and Marshfield Clinic [15]. All study participants provided written consent prior to study enrollment as part of the DNA biobank in each site. Written informed consent was obtained from the next of kin, caretakers, or guardians on behalf of the minors/children enrolled in the study and have been approved by the IRB.

### Study participants

De-identified samples from five different eMERGE sites were evaluated (Table 1). Children and teens, aged through 19 years old composed the pediatric population. For GWAS and PheWAS analyses only those self-reported to have European ancestry and with normal ranges of liver function tests were selected (Table 1), (details described under “Phenotyping”).

**Table 1:** The demographic distribution of EMR-linked eMERGE cohorts.

Cohorts	eMERGE-Sites	#Europeans	M/F	Platforms	Mean age (±SD)
Pediatrics	BCH	148	92/56	Affymetrix-Axiom	13.30 (5.47)
	CCHMC	419	272/147	Illumina-610	
		217	128/89	Illumina-Omni-1	
		184	112/72	Illumina-Omni-5	
		99	12/87	Affymetrix-6	
	CCHMC-Total	919	524/395		11.52 (5.35)
Adults	Marshfield	728	339/389	Illumina660W	
		50	7/43	Affymetrix-6	
	Marshfield-Total	778	346/432		64.9 (11.46)
	Geisinger	691	459/232	Illumina-Omni-Express	70.7 (13.81)
	GroupHealth	657	288/369	Illumina660W	
		101	63/38	Illumina-Omni-Express	
	GroupHealth-Total	758	351/407		67.4 (14.92)
Total		3294	1772/1522		

### Genotyping and imputation

High throughput SNP genotyping was carried out previously in each respective facility as shown in Table 1. Quality control (QC) of the data was performed before imputation. In each genotyped cohort, standard quality control criteria were met and single nucleotide polymorphisms (SNPs) were removed if (a) >5% of the genotyping data was missing, (b) out of Hardy-Weinberg equilibrium (HWE, p < 0.0001) in controls, or a minor allele frequency (MAF) <1%. Samples with call rates <98% were excluded. Recently all eMERGE cohorts have undergone whole genome imputation as described [25]. The imputation pipeline includes SHAPEIT2/IMPUTE2 program and the publicly available 1000-Genomes Project as the reference haplotype panel composed of 1092 samples (release version 2 from March 2012 of the 1000 Genomes Project Phase I, ftp://ftp-trace.ncbi.nih.gov/1000genomes/ftp/release/20110521) [26].

The basic quality controls for eMERGE imputed data provided to us included a threshold of 0.90 for the genotype posterior probability and info score > 0.7 [25]. Info score is the information metric that IMPUTE2 reports [26]. This metric typically takes values between 0 and 1, where values near 1 indicate that a SNP has been imputed with high certainty. This is often used to remove poorly imputed SNPs. Principal component analysis (PCA) was performed to identify outliers and hidden population structure using EIGENSTRAT [27]. The first three principal components explained most of the variance and were retained and used as covariates during the association analysis in order to adjust for population stratification. In addition, UGT1A1 whole exome sequencing data were available to use from the eMERGE-PGx project (a Network collaboration with Pharmacogenomics Research Network (PGRN)) dataset derived from 5163 independent samples from different ancestries [28]. To increase the resolution of the association signal at UGT1A1, this collection was then phased and used as a reference panel for secondary imputation using the SHAPEIT2/IMPUTE2 pipeline described above [26].

### Phenotyping

Levels of serum bilirubin are significantly elevated in a number of diseases. Therefore, we first excluded conditions that either cause overproduction (e.g hemolytic diseases) or decreased in excretion of bilirubin (e.g hepatobiliary diseases). These include ICD-9 codes of 570 to 579.99 with 101 distinct diseases (Chronic liver disease and cirrhosis, Alcoholic fatty liver, drug induced liver disease, gallbladder disease), diagnostic codes of 280 to 289.99 for blood related diseases with 133 categories including hemolytic anemia or Sickle cell disease as well as blood related malignancy with ICD-9 codes of 200 to 209.99 with 391 categories. We also excluded those undergoing chemotherapy or transplant. The same exclusion rules were applied to other liver function enzymes.

Since transient physiologic hyperbilirubinemia is common in the neonatal period, we also excluded children less than one year old. Due to the nature of electronic medical records and since ICD-9 codes, per se, may not be specific and due to underdiagnosed conditions or errors, an additional filtration step was applied in which we excluded samples that still had any out of normal range values (>2 mg/dl) for total serum bilirubin [29,30]. After these filtration steps, the latest lab measures were used for analyses. For other liver enzymes (ALT, ASP, GGT and ALP) and because of wider normal data ranges, natural log-transformations were generated and used for Linear-regression analyses to preserve normal distribution.

A phenome-wide association analysis (PheWAS) was performed in which presence or absence of each PheWAS code that mapped from translated ICD-9 codes were considered as a binary phenotype as described previously [31]. These PheWAS codes were used to define case and control group. Control groups for Crohn’s Disease (CD), for instance, excluded CD, ulcerative colitis and several other related gastrointestinal complaints. The current PheWAS map is available at (http://phewascatalog.org).

### Genome-wide Association Statistics

After performing quality control measures mentioned above, we tested 3,301,391 autosomal SNPs for quantitative association study. Linear-regression analyses assuming an additive genetic model was used on latest total serum bilirubin, ALT, ALP, and AST and GGT using PLINK software package and adjusted by age, sex, principal components, sites and genotyping platforms [32]. In addition, previously known variants associated with serum bilirubin level from the NHGRI catalog were selected as a priori list of 9 autosomal candidate genes and evaluated separately in order to confirm these effects, in which false discovery rate (FDR) methods were used to correct for multiple testing using the Benjamini–Hochberg procedure implemented in PLINK [32]. PLINK was also used for conditional analyses and pairwise SNP-SNP interactions (epistasis) [32]. The ‘‘epistasis” option in PLINK provides a logistic regression test for interaction that assumes an allelic model for interactions and their principal effects in which PLINK makes a model based on allele dosage for each SNP [32]. To graphically display results, LocusZoom and Golden Helix programs were used [33, Golden Helix GenomeBrowse® visualization tool (Version 8.3.0). Bozeman, MT: Golden Helix, Inc. Available from http://www.goldenhelix.com).

## Results

Characteristics of the study participants are presented in Table 1. After exclusions of series of disease conditions described in the Methods, the latest serum bilirubin level and other liver function tests were used for analyses. As shown in Table 1, the mean ± SD age was 67.7±13.40 for adult participants and 12.4±5.43 for pediatrics participants. The mean ± SD of total bilirubin level was 0.56±0.28. All studies showed significantly higher bilirubin levels in males than females, as expected. The mean of total serum bilirubin in males was 0.59±0.31 mg/dl and 0.52±0.26 mg/dl in females. Similarly, it was higher in adults than children (mean± SD of 0.61±0.28 in adults, 0.44 ±0.28 in children). Therefore for all analyses, age- and sex-adjusted analyses (covariates described in Materials and Methods) were conducted.

**Fig 1:**
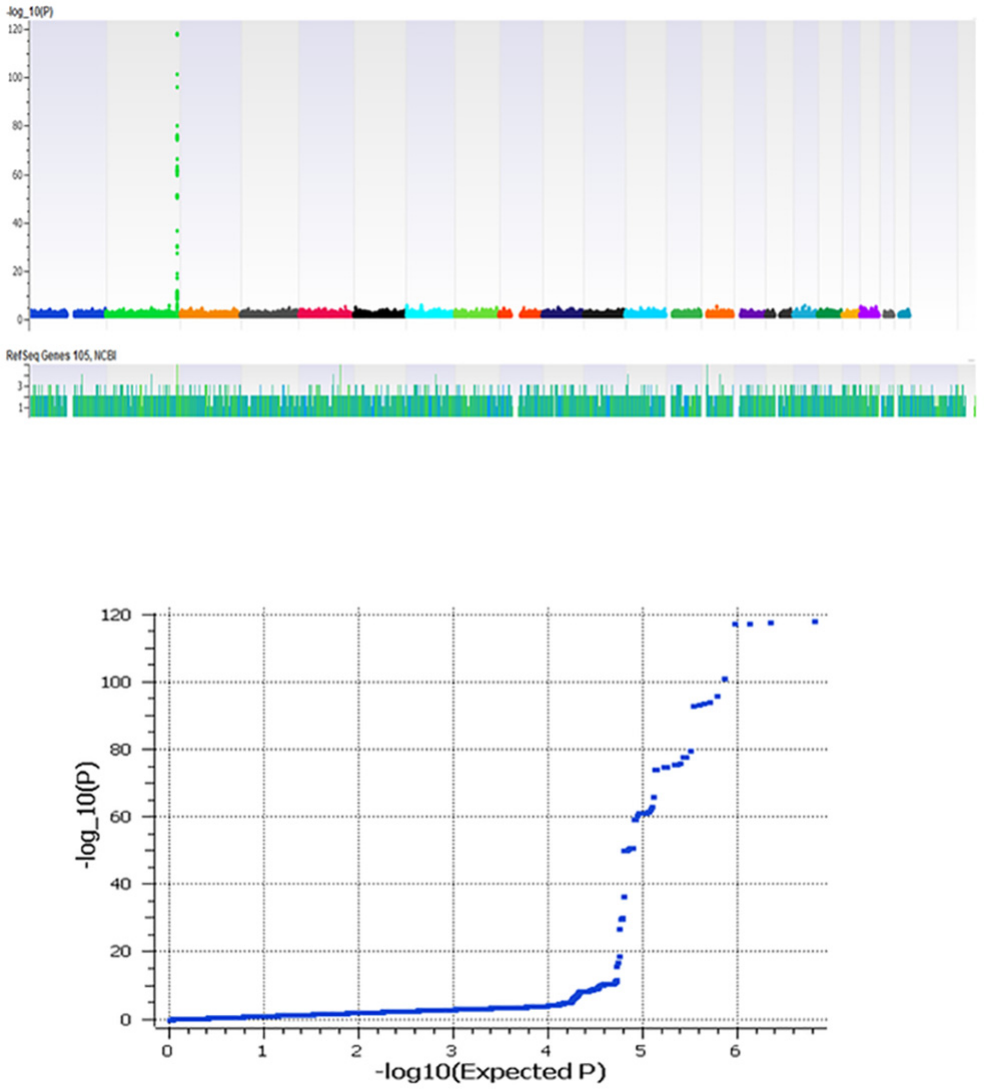
(A and B) Manhattan plot and Q–Q plot of genome-wide markers for total serum bilirubin in 3294 European samples respectively.

Consistent with previous reports, we identified strong genetic signals at *UGT1A1* locus at 2q37. Fig 1 shows the Manhattan plot of this GWAS effect. The overall low inflation rate of (λ = 1.004) was observed. Indeed, the best SNP, rs887829, produced p = 1.30x10^-118^, beta = 0.15. This is equal to an OR = 6.35 when we consider 10% of tail distribution as cases and controls (Table 2). As shown in Table 2, the effect was also strong in the pediatric-only cohort with 1067 participants (p = 8.26x10^-47^, beta = 0.18).

**Table 2:** Quantitative and case-control association result of top SNPs in UGT1A1 for total serum bilirubin levels in pediatric and adult subgroups.

	**Sample size**	**CHR**	**SNP**	**Position** *	**Beta**	**p value** †
Pediatrics	1067	2	rs887829-T	234668570	0.177	8.26E-47
Adult	2227	2	rs887829-T	234668570	0.147	2.00E-63
Total	3294	2	rs887829-T	234668570	0.157	1.30E-118
tail-pheno	Case/Control				**OR**	**p value** ‡
Decile	354/370	2	rs887829-T	234668570	6.347 (5.02–8.01)	8.76E-59

*Map Position: NCBI build 37.

†linear regression association test.

‡Chi-square test.

**Fig 2:**
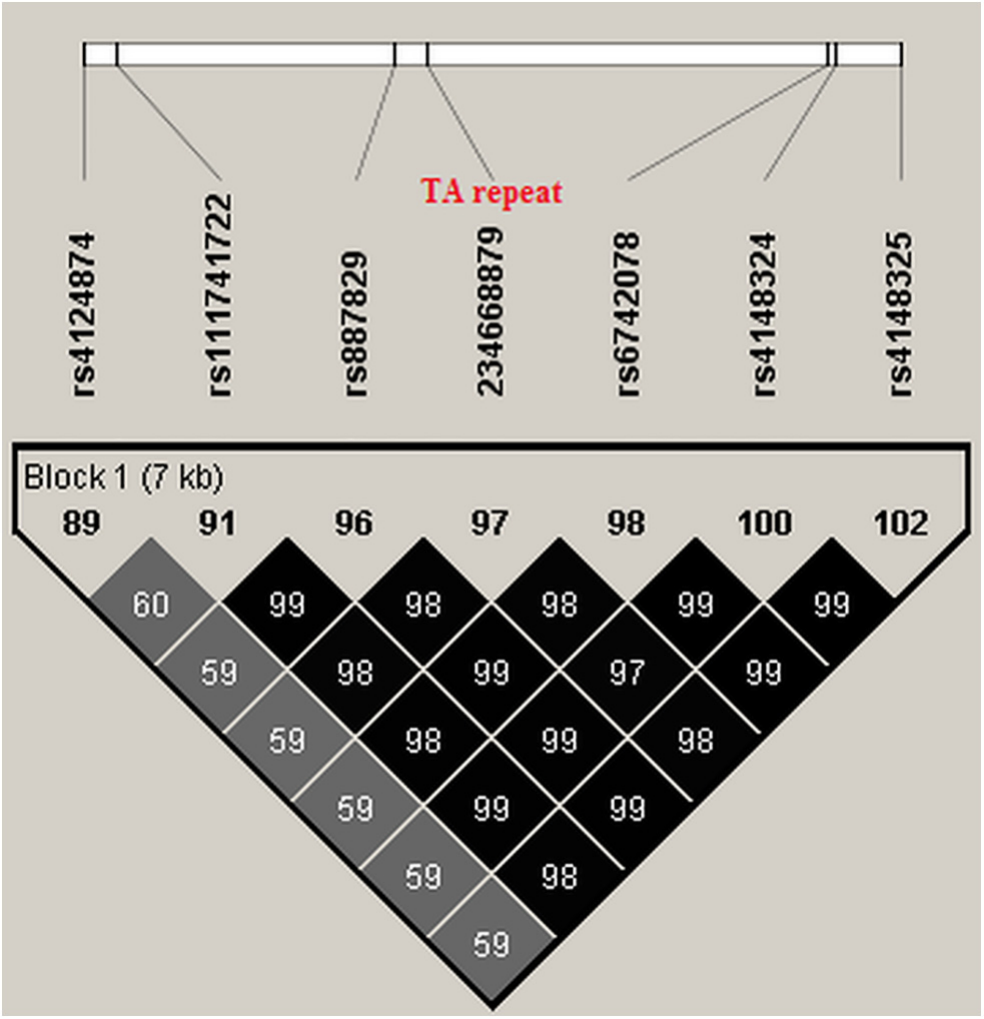
The LD structure between markers in peak association signals. r2 = correlation coefficient as a measure of linkage disequilibrium.

**Fig 3:**
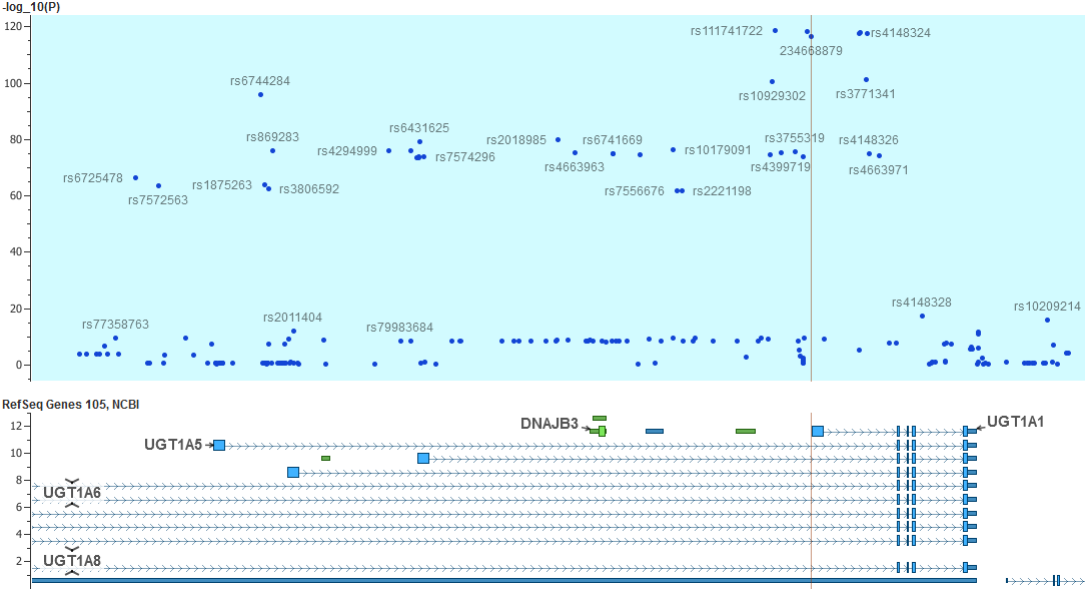
A schematic representation of association map at UGT1A region for total serum bilirubin. The TA repeat location is shown with vertical line.

Next, in order to increase the resolution of this association signal we imputed additional variants using the PGRN sequencing dataset as a reference panel [28]. As a result additional association signals for a number of imputed variants were detected that pass the quality threshold (info score >0.7). In particular, the TA7 indel repeat (rs8175347) that was in linkage disequilibrium with the top marker (rs887829, r2 = 0.99) was imputed and with similar magnitude of effect (p = 9.78x10^-117^, beta = 0.15). Another imputed intronic marker, rs111741722, produced the highest effect in this study (p = 5.41x10^-119^, beta = 0.15). Fig 2 shows the haplotypic LD structure of the TA repeat with nearby markers that all generate similar magnitude of results. Indeed, in this region more than 176 SNPs (or indels) had p<10–8 spanning 150Kb on the long arm of chromosome 2q37.1 (Fig 3). As shown in Fig 2, SNP rs4124874, which also influences gene expression [9] was an incomplete proxy for TA repeat (r2 = 0.60 and produced p = 6.66x10^-75^, beta = 0.12). After conditional analysis controlling for this marker, the TA repeat locus still remained significant (p = 3.58x10^-42^); on the other hand conditioning on the TA repeat diminished significantly all association signal for rs4124874, p = 0.90 suggesting that the TA repeat mainly explains global variations in this region. As shown in Fig 2, TA repeat was in perfect LD with 5 SNP markers (rs887829, rs111741722, rs6742078, rs4148324, rs4148325) spanning less than 10KB, therefore, further conditional analyses were not possible.

### Other effects

Apart from *UGT1A1* effect, no other effects reach a genome significance level (P<10–8) in our cohorts (Fig 1A). There is one other locus at 12p12 (*SLCO1B1-SLCO1B3*) that is consistently reported to be associated with total serum bilirubin at less magnitude [16,17]. In addition, several other genes suggestively have been reported to be associated. We, therefore, more thoroughly evaluated these variants and obtained 9 distinct lead SNPs from the NHGRI catalog and confirmed several previously reported associations for bilirubin at the level of FDR<0.05. These include *SLCO1B1-SLCO1B3*, *TDRP and ZMYND8* (Table 3). SLCO1B1 is an organic anion transporter (OATP1B1) important for uptake of bilirubin in liver. The nonsynonymous SNP, rs4149056 V174A, which alters uptake activity of this transporter produced p = 0.0004, beta = 0.03 in our combined cohorts (Table 3). In this region SNPs located at *SLCO1B3* was also associated including rs10841661 (Table 3). There was a 350Kb distance between these two family members of B1 and B3 with r2 = 0.002. Therefore, conditional analysis controlling for rs4149056 still shows association effects at rs10841661 (p = 0.0008) suggesting an independent effect in *SLCO1B3*.

**Table 3:** Other effects associated with total serum bilirubin at FDR<0.05.

CHR	Gene	SNP	BP	Minor allele*	BETA	P value	P value- Adjusted for rs887829	P value- Interaction with rs887829	REF
8	TDRP	rs17665859	445601	C	0.037	0.003	0.009	0.28	17
12	SLCO1B3	rs10841661	20984832	T	0.025	0.0003	0.0006	0.24	20
12	SLCO1B3	rs17680137	21015906	G	0.029	0.001	0.0013	0.75	20
12	SLCO1B1	rs4149056	21331549	C	0.032	0.0004	0.00009	0.21	16
20	ZMYND8	rs7262634	45834279	C	0.042	0.005	0.0192	0.2	24

Conditional P values adjusted for lead SNP in UGT1A1 (rs887829) as well as pairwise SNPxSNP interactions with (rs887829) are shown.

*All effects are for minor alleles.

We next tested for any effect of the *UGT1A1* gene on the association of bilirubin levels with these genes and SNP rs887829, the top marker on the *UGT1A1* locus in the model, was used for conditional analyses. As shown in Table 2, no association disappeared after controlling for rs887829, suggesting that part of the variability of total serum bilirubin can be explained by these other genes. Furthermore, although no significant gene-gene interaction was observed, some association improved (Table 3).

### Rare effects

In the *UGT1A1* gene, there are more than 50 known stop codon, gain of function or frame shift mutations reported in families with CN-type 1 or 2 [34]. These mutations are extremely rare and homozygosity is link to autosomal recessive early lethality especially in CN type I. In PGRN sequencing data we identified 16 of these variants in 44 participants but all were carriers (heterozygous); Serum total bilirubin were available for 9 of them that all were within normal range consistent with a recessive effect. No homozygote recessive or compound heterozygote participants were detected.

As mentioned above the SNP rs4148323 (G71R) mutation is more common in the Asian population. This variant is associated with a milder form of hyperbilirubinemia and Gilbert’s syndrome. PGRN sequencing data revealed two Asian participants that were homozygous for coding SNP rs4148323 (G71R). The means of total serum bilirubin for these participants were 1.52 (95% CI = 1.032–2.018) and 0.85 (95%CI 0.68–1.0) respectively. They have not been diagnosed clinically with jaundice according to EMR, nor did they have any risk alleles in TA repeat, rs4124874 or any other detected rare variants.

### Other Liver function test

**Fig 4:**
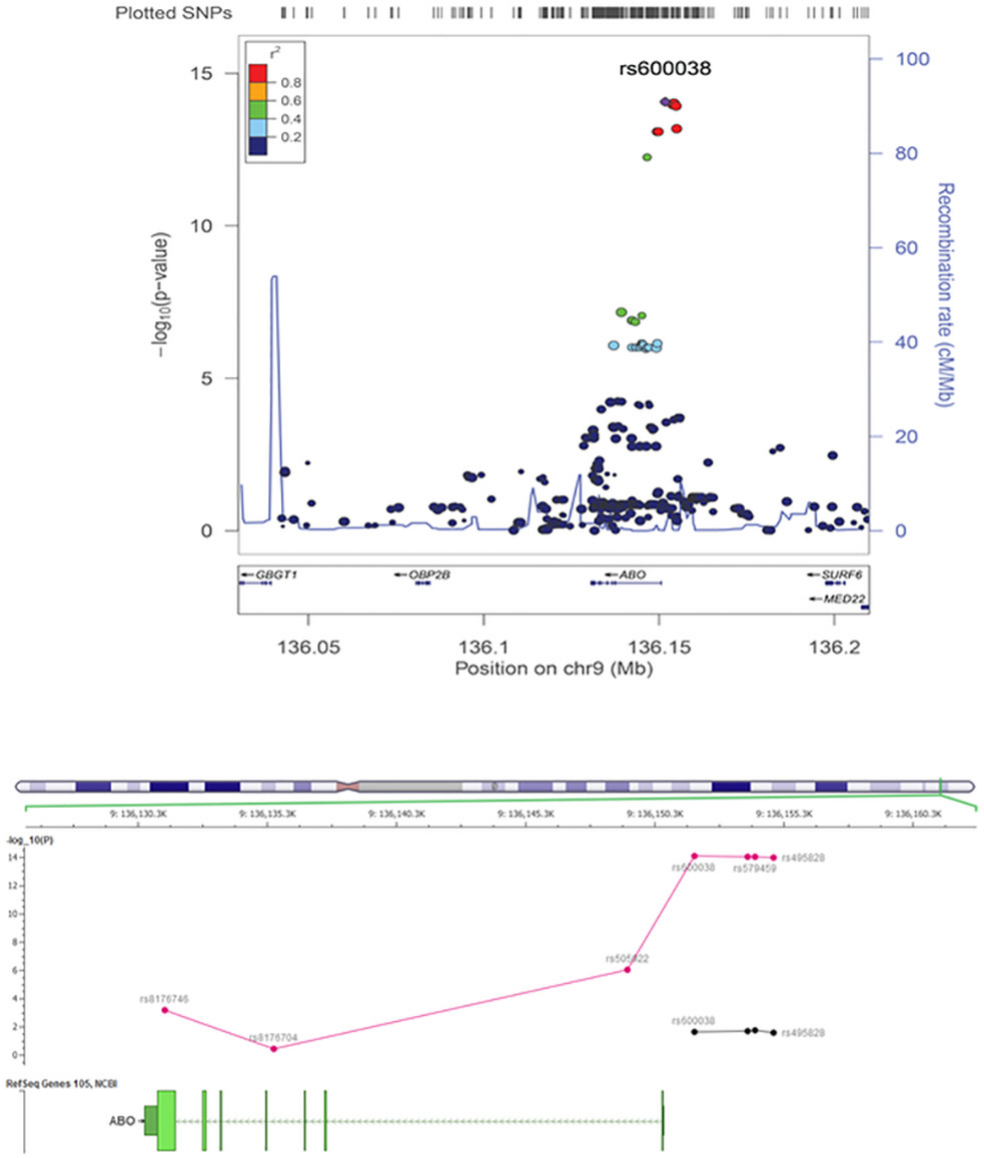
(A) The association signal between total serum ALP and the ABO blood locus. (B): The association of lead SNPs at the ABO locus with serum ALP and their relation with ABO tag SNPs (rs8176746, rs8176704, rs505922); A significant drop in association effect in lead SNPs after controlling for ABO-tag SNPs are shown (red and black dots represent before and after conditional analyses respectively).

Except for serum total alkaline phosphatase, no other liver enzymes produced a genome wide significant effect (P<1x10^-8^). The effect of the ABO blood group locus and total alkaline phosphatase that has been replicated in different studies was confirmed in our study (best p ≤1x10^-15^ for SNP rs600038, Table 4, Fig 4A). The effect was consistent across pediatric and adult cohorts. ABO blood type is known to be correlated with serum alkaline phosphatase, therefore we next identified perfect surrogate SNPs tagging *ABO* alleles in our dataset which include: rs8176704 for the A allele, rs505922 (or rs612169) for the O allele and rs8176746 (or rs8176672) for the B allele [35,36]. As shown in Fig 4B, after controlling for all three *ABO* SNP determinants, the association signal dramatically reduced for the lead SNP rs600038 with p-conditional = 0.02, consistent with previous studies. Of note, the linkage disequilibrium between lead SNP rs600038 and three *ABO* SNP determinants (rs505922 and rs8176746, rs8176704) were r2 = 0.39, 0.03 and 0.01 respectively.

**Table 4:** 

Enzyme	Gene	CHR	SNP	BP	Minor allele	BETA	P value	REF
ALP	ABO	9	rs651007	136153875	T	-0.034	1.06E-14	22,23
	ABO	9	rs600038	136151806	C	-0.034	8.608e-15	22,23
	ABO	9	rs579459	136154168	C	-0.034	9.436e-15	22,23

### PheWAS approach

To explore pleiotropy of the TA-repeat in UGT1A1 for other clinical diagnoses, we performed a PheWAS study in adult and pediatric cohorts separately using available ICD-9 codes as previously described [31]. As expected, since we excluded all participants with hyperbilirubinemia and related conditions, we did not find any associations for these conditions. There were 377 distinct diagnostic codes for adults with minimum of 100 participants in each category. After correcting for multiple comparisons, no results remained significant. The best overall effect was a trend toward associations with cerebrovascular disease and ischemic stroke in adult-only cohorts adjusting for age, gender, site, platforms and PCs (unadjusted p = 0.0008, adjusted p = 0.056, OR = 0.75, 95%CI 0.63–0.88). There were 464 cases with this diagnosis and 1433 controls. Of note this was a protective effect for the TA7 repeat. In addition, results for other atherosclerosis-related conditions such as ischemic cardiovascular disease were in the same direction but only suggestive (data not shown). The number of samples for these conditions were small in pediatrics cohorts (<10).

## Discussion

In this GWAS analysis from five eMERGE sites we confirmed the substantial contribution of *UGT1A1* on human serum bilirubin levels and a number of other transporter genes, including *SLCO1B1* and *B3* that influence variation in bilirubin levels. This is the largest GWAS study for serum bilirubin in a pediatric cohort. We found that this effect is strong in pediatric as well as adult populations (Table 2). Further imputation using PGRN sequencing data revealed that the TA indel repeat (UGT1A1*28, rs8175347) is in strong LD with 5 additional SNPs (r2>0.98) and mainly responsible for the GWAS signal at this location. Indeed, this locus by itself explained 14% of total variations of serum bilirubin in our cohorts comparable with previous studies [16]. In comparison only 0.2% of total variation of serum bilirubin can be explained by rs4149056 SNP in *SLCO1B1* in our cohort. Similar to previous studies, we did not detect a significant gene gene interaction between *SLCO1B1/B3* and *UGT1A1* (p = 0.21, Table 2) [16]. We confirmed a number of other effects as shown in Table 3 including *TDRP* and *ZMYND8*. We had 90% power to detect such association in our studies (average MAF = 0.10, beta 0.03–0.05, OR = 1.1–1.2).

We applied a successful strategy using electronic medical records to identify individuals with normal variations in serum bilirubin and other liver function tests by excluding more than 300 blood and liver-related diseases and conditions (see method). Electronic health records have enormous phenotypic heterogeneity. There are many reasons that a patient may have hyperbilirubinemia that range from a simple viral hepatitis to hemolytic crises due to medications. Including serum bilirubin for these non-genetic conditions may produce noise and skew the normal distribution of the quantitative phenotype under study. Obviously additional studies are necessary to address any disease specific condition. Similarly we applied stringent quality criteria and thresholds for genotyping data and adjusted all results by age, sex, site of study, platform and principal components. We evaluated the eMERGE PGRN dataset for rare variants at *UGT1A1* and as a result we identified two Asian participants with homozygous for the rs4148323 minor allele resulting in a (p.Gly71Arg) coding change. Interestingly they did not have additional risk alleles in the TA repeat or rs4124874 variants suggesting an independent effect for (p.Gly71Arg). One of these participants shows a subclinical hyperbilirubinemia (latest total bilirubin = 2.1 mg/dl) possibly associated with the effect of this missense variant [37]. As mentioned above, this variant is rare in other ancestries. Other available liver function tests were normal in these two participants. In addition, we identified 44 heterozygote (or carrier) participants with other potential deleterious mutations such as (rs34993780, p.Tyr486Asp). Since homozygous variants are extremely rare in population (1:1000000) with severe early hyperbilirubinema (CN typ1 or 2), we did not expect to find these homozygotes in our population panel cohorts. Interestingly no compound heterozygote was detected.

Among other liver function tests, we confirmed the effect of the *ABO* locus on serum alkaline phosphatase. This effect has been consistently replicated in other studies [22,23]. Approximately 90% of serum alkaline phosphatase (ALP) originates from liver, bone and kidney, while 10% from intestine and 1% from placenta [38]. Most of the intestinal ALP is attached to ABO antigens on the surface of erythrocytes by a glycosyl-phosphatidylinositol anchor, however, with different binding capacity depending on different blood groups. Erythrocytes of blood type A bind to almost all intestinal ALP, while erythrocytes of blood type B/O bind to a much lesser degree. This results in an increase of intestinal ALP in serum of individuals with blood type B/O [39]. The observation that association between rs600038 and serum ALP dramatically reduced after adjusting for ABO-related variants support this physiologic process; although a residual effect exists (p = 0.02, Fig 4B) that may indicate other regulatory roles of this locus for serum alkaline phosphatase or underlying confounding effects since the *ABO* locus is associated with a variety of metabolic related phenotypes including serum levels of intercellular adhesion molecule-1 (ICAM-1), E-selectin, and P-selectin that participate in inflammatory processes by promoting adhesion of leukocytes to vascular wall endothelium, effects on low-density lipoprotein and total serum cholesterol levels as well as coronary artery disease and stroke [35,40,41].

The major strengths of our study include careful quality control and minimal population stratification. Electronic medical records, however, have several study limitations including human errors in extraction of lab measurements or errors in billing code and clinical diagnosis. In addition the population under study may have wide spectrum of different diseases and therefore cannot represent a random sample from the normal general population. We have controlled all relevant medical diagnoses in the analyses to avoid potential sampling bias. The associations with serum bilirubin levels and *UGT1A1* were in fact highly consistent with previous publications that often recruit well-characterized participants.

Total serum bilirubin is associated with several clinical outcomes, including cardiovascular disease, diabetes and drug metabolism that warrant additional study. In fact, bilirubin is a potent antioxidant and higher levels of serum bilirubin may offer a therapeutic advantage in oxidative stress-mediated conditions [42]. In this context, we detected a suggestive protective effect of TA7 repeat against chronic cerebrovascular disease that point to this direction (OR = 0.75). Our adult cohort, consist of elderly participants with the mean age of 67.7 (95% CI 63.00–72.33) that could well-represent a long term sequela of bilirubin on vascular system. Obviously excluding potential liver disease patients in this study was a key step to clarify the inverse associations between bilirubin and stroke as discussed in other published works [43]. Finally, susceptibility to severe neutropenia following the administration of Irinotecan in TA7 homozygotes with colon cancer is particularly interesting and this data will be used for future pharmacogenetic studies across the eMERGE network.
